# Promises and Pitfalls of Internet Search Data in Mental Health: Critical Review

**DOI:** 10.2196/60754

**Published:** 2025-02-18

**Authors:** Alexandre Andrade Loch, Roman Kotov

**Affiliations:** 1 Laboratory of Neuroscience (LIM-27), Institute of Psychiatry, University of São Paulo, Rua Dr. Ovidio Pires de Campos, 785, 4th floor, room 4N60, São Paulo, 05403903, Brazil, 55 11996201213; 2 Renaissance School of Medicine, Stony Brook University, New York, NY, United States

**Keywords:** privacy, stigma, online, prevention, internet, search data, mental health, health care, clinical information, World Health Organization, WHO, digital health, mental illness, digital technologies, social network, mobile health, mHealth

## Abstract

The internet is now integral to everyday life, and users’ web-based search data could be of strategic importance in mental health care. As shown by previous studies, internet searches may provide valuable insights into an individual’s mental state and could be of great value in early identification and helping in pathways to care. Internet search data can potentially provide real-time identification (eg, alert mechanisms for timely interventions). In this paper, we discuss the various problems related to the use of these data in research and clinical practice, including privacy concerns, integration with clinical information, and technical limitations. We also propose solutions to address these issues and provide possible future directions.

## Introduction

The internet is now integral to everyday life, and the world’s population has never been more interconnected. Therefore, the World Health Organization (WHO) launched a global strategy in 2020, proposing a road map to link the latest developments in innovation and digital health to improve health outcomes. This initiative was of critical importance, given the recent studies suggesting an increase in prevalence rates of mental disorders after the COVID-19 pandemic [1], and internet and other digital technologies demonstrating a great potential to reverse this trend [2].

The internet is one of the most important sources of information, fueled by increasingly powerful search engines that link users to websites. Each day, approximately 8.5 billion searches are performed using Google alone [3], and these extensive internet search data could be of strategic importance in mental health care. Search terms may reflect an individual’s curiosity, desire for information, emotional well-being [4], mental health state [5], or need for assistance [6,7]. In this context, internet search data could be of great value for the early identification of mental health disorders and for facilitating pathways to care, reducing the long-standing delays in seeking help and improving outcomes.

## Methods

### Overview

A critical review was conducted with the aim of capturing the available evidence on the use of search engine data in mental health research, specifically in studies investigating the link between individual users’ mental health states and their internet search data. In line with previous studies in the field, search terms included a combination of search engine–related terms (“internet search” OR “google search” OR “bing search” OR “internet search data” OR “search engine*”) and terms addressing mental illnesses (“psychiatric disorder*” OR “mental disorder*” OR “mental illness*” OR “depressi*” OR “anxi*” OR “psychos*” OR “psychotic” OR “schiz*” OR “bipolar”). The search was conducted in the following databases: PsycINFO, PubMed, and Web of Knowledge. Inclusion criteria comprised studies that examined the relationship between internet search data at the individual level and subjects’ mental states. There were no language or publication time restrictions. The exclusion criteria included reviews and strictly theoretical articles without original data. Poster presentations, grey literature, and unpublished transcripts were also excluded.

A total of 1184 publications were identified in the initial search (duplicates excluded). The authors screened the titles and abstracts of all papers, identifying 87 relevant papers. These 87 relevant papers were read in full, with 9 meeting the criteria in the final round, for analysis of individual search data. Additionally, 46 studies examined the use of internet search data at the population level (eg, Google trends) and will be partly addressed in this paper.

### Population-Level Research

Internet search data may provide valuable insights into users’ mental state. Recent studies suggest that signals from search history and YouTube data may be correlated with depression and anxiety levels [8], predict suicidal behavior [9] and diagnoses of schizophrenia spectrum disorders [10].

For example, one study observed that individuals with mood disorders conducted internet searches significantly more frequently than those with schizophrenia spectrum disorders from 6 PM to midnight and used significantly more words related to negative emotions compared to healthy volunteers [10]. Internet help-seeking may exhibit daily fluctuations, as evidenced by describing a significant rise in depression-related search query volumes toward the evening and night [11,12]. Other studies observed that information-seeking on Google related to major mental disorders terms followed seasonal patterns similar to those found in seasonal affective disorders [13]. Peak searches generally occur over the winter months, with troughs occurring during summer [14]. Interestingly, in a study encompassing 54 geographical areas worldwide, internet searches for depression were influenced by seasonal changes in higher latitudes but not in tropical areas [15].

Many researchers also used internet search data to examine population mental health trends. Wang et al [4] developed a public opinion dictionary using emotionally enriched texts extracted from social media. They combined the public opinion dictionary with a word vector model and internet search trends to develop a composite anxiety and depression index for specific regions over time. Ford et al [16] collected data on the frequency of Google searches for 15 affect-related terms, and paired them with data on health, self-reported emotions, psychological well-being, personality, and Twitter postings. Several internet search scores were correlated with depression, self-reported emotions, and well-being. An interesting study analyzed the association between the Oklahoma earthquakes and anxiety-related Google search episodes [17]. For each additional earthquake with a magnitude of 4 or higher, the proportion of Google anxiety-related search episodes increased by 1.3%, with 60% of these increases persisting for a month. A similar trend for increased anxiety-related search terms was observed after an earthquake in Japan [6]. These findings suggest that internet search data could be a valuable tool for estimating regional or country-level prevalence of mental health disorders [18].

In line with the disaster-related findings, a recent topic of investigation has explored the interplay between the COVID-19 pandemic, mental health, and internet search data [19]. In a study involving 50 countries, Bing search query data and Google Trends search volume data identified a significant increase in the volume of the most-searched anxiety-related themes in 2020 [18]. Many other studies also observed an increase in depression-related search terms during the pandemic [20,21]. An important study combined cellular mobility tracking, online search data, and Google Trends search volumes for terms related to economic stress, mental health, and suicide in the United States [22]. The authors observed that pandemic-related isolation coincided with acute economic distress, which may be a risk factor for poor mental health and suicidal behavior. Fluctuations in mental health have been associated with lockdown stringency [23,24], vaccine discovery [25], and number of COVID-19–related deaths, as inferred from search data volume. A systematic review conducted in 2021 observed a significant increase in search terms related to mental illnesses during the COVID-19 pandemic [26]. This link was confirmed by analysis associating the number of COVID-19 cases and deaths with internet search volume for specific keywords, as well as by content and sentiment analysis. Given the increasing interest in this topic, particularly after the pandemic, internet search data has been proposed as real-time surveillance tool for population mental health [27-31]. However, evidence linking search data trends to population mental health remains inconclusive. A study conducted in the United Kingdom found that Google Trends data were not a useful indicator of changing population mental health levels during a public health emergency [32]. Nevertheless, the authors acknowledged that it may serve as an indicator of loneliness.

At last, internet search data has been increasingly used in suicide research. Many studies showed that specific search terms were correlated with increased suicide rates at the population level [33-39]. While most of these terms are intuitive (eg, “anxiety disorder,” “laid off”) [33], others are less obvious (eg, “allergy”) [34], highlighting the need for further culture- or region-specific studies. For instance, suicide is a complex phenomenon influenced by a variety of psychosocial, biological, environmental, economic, and cultural factors. Regarding internet searches, there has been significant effort toward responsible reporting to prevent suicide contagion [40], for example, when celebrity suicides are announced [41]. The well-known Werther effect could also be traced through internet search data, as shown by increases in suicide-related information searches after celebrity suicides, such as those of Robin Williams [42] and Kimi Qiao [43]. Ideally, search data analysis should take into account such societal factors and other socioeconomic data (eg, unemployment rate) [44] to enhance suicide prediction and facilitate timely public health measures.

### Individual-Level Research

Search data are not limited to search terms used; it may also include time stamps, location history, and YouTube search data and videos viewed—available through platforms such as Google Takeout [8]. Consequently, it may offer a diverse and rich data source at the individual level for mental health research, potentially offering insights into an individual’s behavior, thoughts, and concerns [45]. The content of these searches can be linked to clinical data obtained from electronic health records or direct patient assessments to identify clinically relevant language. Studies may focus on searching key words (eg, “voices” or “psychiatrist”), categories of words (eg, negative emotion lexicons), composites of words and phrases derived by machine learning, or contextual embeddings identified by deep learning models [46]. Despite this potential, there is a discrepant paucity of data on individual-level studies compared with the abundance of population-level internet search data investigations, for reasons addressed further in the text.

Among the few studies analyzing individual-level information, one enrolled 108 college students to link their Google search data to mental health data obtained from a self-administered mental health questionnaire [8]. The authors showed that individual-level search history could provide identifiable signals for detecting low self-esteem with reasonable accuracy. Another study of 92 adults, which aimed to develop an online automated screener, found that online behavior can help create automated classification tools for loneliness with high accuracy [47]. Kirschenbaum et al [48] requested 20 individuals hospitalized for psychosis to provide access to their Google archive data regarding text and timing of search queries. They observed that individuals with early psychosis appeared to use the internet for obtaining information about their early symptoms and experiences prior to their first contact with psychiatric care. More recently, Milton et al [49] successfully provided information on user’s help-seeking behavior for common mental health disorders such as depression and anxiety based on the search terms. In another study, search queries from 105 individuals with schizophrenia spectrum disorders, mood disorders, and healthy volunteers were analyzed, using one year of data prior to the first psychiatric hospitalization [50]. Significant differences were observed one year in advance, in the timing, frequency, and content of search activity in individuals with mental illnesses compared with healthy volunteers. Additionally, 43 patients hospitalized for suicidal thoughts and behaviors granted access to their search logs from the 3-month period preceding psychiatric hospitalization [33]. Suicide-related searches included queries related to the television show 13 Reasons Why—a popular series about a teenage girl who dies by suicide that was airing during the study’s recruitment period—as well as other suicide-related topics in the news media. Notably, some participants searched for highly specific and unusual suicide methods; one participant conducted numerous searches related to self-inducing cancer and investigated the possibility of purchasing live cancer cells on web-based platforms.

Due to this high-quality information and the potential for timely intervention for a serious event, search engines trigger help information after suicide-related searches. Nevertheless, caution should be taken as all that glitters is not gold. A study conducted by Haim et al [51] examined this complex topic of searching for suicide-related information. While investigating whether suicide-prevention results (eg, a helpline box) appeared after internet searches for suicide-related information, the authors showed concerns about possible ambivalent and potentially detrimental algorithmic decision-making. In their study, the suicide-prevention information (ie, the helpline box) was displayed only to a limited number of search agents searching for harmful suicide-related information.

Overall, internet search data might offer real-time alerts and opportunities for timely and early interventions, potentially improving mental health outcomes. Most importantly, it may be a useful tool to address the delay in seeking help for mental disorders [52]. Researchers are also investigating whether it could potentially be used as an adjunct to ongoing clinical management, such as relapse prevention in psychosis [10], with a small number of pilot studies currently underway. While internet search studies show potential, the existing data remain limited to a few studies with small sample sizes, and several ethical and methodological concerns exist related to these studies.

### Problem Statement

One of the most important issues concerning the use of internet search data for mental health care research is privacy [53]. Uncertainty about users’ willingness to share data is particularly critical across diverse groups and countries, raising concerns about the feasibility of large-scale data collection and potential biases in the data obtained [53]. Additionally, since the recent privacy violation lawsuits that gained media attention in the United States, an atmosphere of distrust has been created in the last few years posing a challenge that needs to be acknowledged and overcome [54]. In this context, addressing consent issues from vulnerable populations such as children and older adults is complex, and requires careful consideration of ethical guidelines and legal frameworks.

The second problem involves integration of internet search data with other sources of information such as electronic health records by researchers. Besides the privacy concerns mentioned, this process poses several challenges, which should be addressed from an interdisciplinary perspective. Storage and processing of large volumes of sensitive data could generate barriers for research teams. Computer scientists, data managers, and privacy officers should address these issues and define secure pipelines for information transfer—for example, defining how information should be extracted from internet and health records and aggregated into a secure and anonymized database available to the research team. Further, interpreting internet search patterns and clinical data requires advanced data analysis methods and interdisciplinary collaboration to avoid producing black box findings [55].

Third, additional general technical challenges must be addressed. For instance, ensuring user-friendly procedures for giving permission to access data, accounting for potential changes in search behavior due to awareness of monitoring (which could impact data quality and reliability), determining ownership of shared resource data, especially in regions where phones are communal, and addressing digital and mental health literacy. Perceived risk related to stigma and adherence to digital mental health initiatives are also important considerations.

Fourth, existing studies have been limited by small sample sizes—typically 100 participants or fewer. However, for accurate detection of psychopathology, language models must be highly complex and training of such models requires thousands of observations [46]. Moreover, model generalizability must be evaluated using new sample populations.

Finally, as scarce evidence exists regarding the use of internet search data for mental health care, the clinical accuracy and utility of identifying people at risk, diagnosing conditions, and reliably monitoring changes in symptoms remains to be determined. Retrospective data may have limitations in predicting future outcomes effectively (eg, data leakage, model overfitting), raising concerns about its reliability for certain types of analyses and predictions. Researchers should acknowledge that the clinical utility of information that can be extracted from internet searches is currently unclear.

### Proposed Solutions and Implementation

Addressing privacy concerns and distrust requires targeted actions. First, a thorough ethical analysis of research initiatives, coupled with clear and up-to-date forms of participant consent, is warranted [56]. In this context, it is important to reassure the participants that collected data will be used only for health research and not for commercial purposes [56]. Modern data security techniques, including deidentification of data, should be employed to ensure privacy. Additionally, to improve trust in data sharing, initiatives should be led by reputable academic institutions, with big technology companies serving as facilitators or in other supporting roles.

To address the second issue of integration of data, we propose to conduct research in large hospitals and universities, where considerable amount of clinical data are available to support large sample size. Inclusivity and diversity should be prioritized by involving academic institutions in and beyond North America and Europe—where most scientific evidence is generated—to include low-and-middle-income countries. Adequate funding should be designated to such projects to build a team of experts and to acquire enough hardware and software to deal with large and heterogeneous datasets. Consortia to facilitate the exchange of experience and knowledge between teams in the academic and commercial fields should be encouraged.

Third, technical challenges need to be addressed through rigorous study designs. Most importantly, the final models should be evaluated in previously unseen samples and rigorous assessment of overfitting, given the large quantities of data. The user-friendliness of the data-sharing procedures should be investigated by studies on usability and acceptability. The Hawthorne effect must be considered in study designs, and the possible use of ecological (or retrospective) data is warranted. Participant consent for sharing this sensitive data requires detailed discussions with researchers, especially regarding data ownership. Finally, as with all mental health initiatives, stigma needs to be addressed by increasing mental health literacy and providing accurate information on mental illnesses. This will ultimately stimulate participation in the studies and in theory, reduce possible biases (Table 1).

The challenge of stigma and privacy concerns from a broader perspective should also be addressed based on the proposed solutions. Additionally, other actions aiming at regulating the use of internet search data at the clinical level should be proposed. These legal actions should protect the society against inadequate commercial exploitation of technology, technology misuse, breach of confidentiality, and related issues. Together with campaigns to improve mental health literacy and to decrease distrust in big data collection and its use for medical purposes, public actions to support the consortium between the academia and companies such as Google, and government sectors should be stimulated and publicized. This environment of multiple cooperation between diverse sectors may encourage population participation and technology adoption. Further, enrollment of lived experience in all processes of research (from design until governance) may further improve public adoption and acceptability of new technologies.

Finally, diversity of samples is needed to maximize representativeness and generalizability. For instance, unequal access to the internet among populations could introduce a selection bias. The identified patterns should be investigated for multicultural reliability; cutting-edge and ever-evolving data analysis techniques will increasingly improve the performance of technologies. To establish clinical utility, models should be tested in real-world scenarios to demonstrate their superiority over currently used screening strategies and clinical evaluations. Accordingly, implementation feasibility of the clinical context needs to be assessed. From a patient perspective, their role in generating, communicating, and annotating data must be tested and defined. From the clinical practitioners’ view, acceptability, the issue of how to integrate these data in their decision-making, and communication regarding the use of search queries within clinical practices need to be investigated.

**Table 1.** Summary of problems and proposed solutions for digital technology in mental health research.

Problems	Proposed solutions
Privacy concerns	Ethical analysis, participant consent, data securing, research led by academia
Data integration	Enroll large clinical datasets, funding for multidisciplinary team
Technical challenges	Sufficient training data (usually thousands of observations) and evaluation in new datasets
Clinical accuracy and utility	More diverse samples, different sociocultural settings, include patient samples and outcomes of clinical interest (eg, prognosis, response to specific treatment)

## Conclusion

Internet search data that are integral to daily life generate large amounts of individual data and have the potential to revolutionize mental health care. In summary, individual search data might be of great value in (1) the prediction of mental health problems using search history, (2) real-time identification and development of alert mechanisms to target timely interventions, and (3) adjuncts to clinical management. However, ensuring participants’ trust and confidentiality is essential. Consortia between academic institutions and technology companies should be encouraged, aimed at increasing the population’s reliance on these data, knowledge exchange between different sectors, and big dataset handling. Further research is strongly encouraged to address feasibility and acceptability. Existing studies are too small to ensure replicable results; future studies should involve significantly larger sample sizes. New initiatives should be inclusive and diverse, incorporating perspectives from the Global South to ensure the universality of findings.

## Abbreviations

WHO: World Health Organization
